# Perception of sexual harassment among female athletes participating in team sports in the Northern Cyprus

**DOI:** 10.3389/fpsyg.2025.1676528

**Published:** 2025-10-29

**Authors:** Deniz Erdaǧ, Mehmet Özeriç, Bingül Harmancı

**Affiliations:** ^1^Faculty of Sport Sciences, Coaching Education Department, Near East University, Nicosia, Cyprus; ^2^Faculty of Art and Sciences, Psychology Department, Near East University, Nicosia, Cyprus

**Keywords:** sexual harassment, female athlete, team sports, gender issues in sport, sport psychology

## Abstract

**Introduction:**

Gender inequality in sports manifests in various forms, including differences in treatment, expectations, and exposure to inappropriate behaviors. Female athletes often face subtle and overt forms of discrimination, particularly in relation to coach behavior and sexual harassment. Understanding how female athletes perceive and react to such behaviors is crucial for developing effective strategies to foster safer and more equitable sports environments. This study aims to examine the multidimensional nature of gender inequality in sports by exploring female athletes' perceptions of coach behavior, their reactions to sexual harassment, their ambivalent sexist tendencies, and their attitudes toward sexual harassment.

**Methods:**

This research utilized a quantitative survey design. Data were collected through the Socio-Demographic Information Form, the Reactions to Sexual Harassment in Sports Scale, the Ambivalent Sexism Inventory, and the Attitudes Toward Sexual Harassment Scale. The Coach Behavior List was also administered to assess athletes' perceptions of coach conduct. Participants included female athletes from various sports disciplines. Statistical analyses were performed to identify differences across age groups and sports disciplines.

**Results:**

The findings indicated no statistically significant differences in the total and subscale scores of the Coach Behavior List and the Reactions to Sexual Harassment in Sports Scale across different age groups. However, significant differences emerged across sports disciplines. Handball players reported lower scores on the sexual behaviors and non-instructional threatening behaviors subscales, suggesting reduced exposure to these forms of coach misconduct. Additionally, football and basketball players demonstrated stronger emotional reactions to sexual harassment compared to handball players. These patterns indicate variability in gender dynamics and exposure to harassment across different sports.

**Discussion and conclusion:**

The results highlight how gender inequality in sports is shaped not only by individual factors but also by the cultural and structural norms of specific disciplines. The lower levels of reported misconduct among handball players may reflect differing power dynamics or gender norms within that sport. Conversely, the stronger emotional reactions observed among football and basketball players suggest heightened sensitivity or exposure to harassment. These findings emphasize the need for multilayered interventions aimed at raising awareness, empowering female athletes, and addressing sexual harassment through prevention and education initiatives. Creating safer and more inclusive environments in sports requires both institutional reforms and cultural change.

## Introduction

Sexual harassment stands out as a serious social issue that female athletes frequently encounter in sports settings. While the Turkish Language Association defines harassment as “causing discomfort,” sexual harassment involves sexual content that violates an individual's personal boundaries. This encompasses sexually explicit remarks, unwanted physical contact, inappropriate gazes, digital harassment, and gender-based discriminatory expressions (Yoon et al., 2020).

The World Health Organization (2022) defines sexual violence as any sexual act or attempt to obtain a sexual act without the individual's consent, emphasizing that such acts are often used as tools of power, pressure, and control. Among the various forms of sexual violence experienced by female athletes, exploitation, coercion, and a culture of silence, particularly within coach-athlete relationships, are particularly concerning (World Health Organization, 2022).

Due to their hierarchical structure and the physical proximity they require, sports environments can become high-risk areas for women. There are documented cases in which coaches have exploited these power dynamics, particularly targeting young female athletes motivated by career aspirations, scholarships, or national team selection opportunities (Kavanagh et al., 2023).

For instance, the case involving Larry Nassar, the former physician of the U.S. Women's Gymnastics Team who sexually abused more than 300 athletes, was not only treated as an individual crime but also as a systemic failure on the part of sports institutions to fulfill their responsibilities (Denhollander, 2020). Similarly, the revelations of British footballer Andy Woodward exposed how years of abuse had been concealed due to the culture of silence in football. These cases illustrate how coaches may abuse their authority for sexual gain (Kerr et al., 2020).

Examples of sexual harassment faced by female athletes include unnecessary physical contact during training, invasion of privacy in locker rooms, sexist comments about appearance, and sexually suggestive messages sent privately (Johansson et al., 2022; Vertommen et al., 2017; Timpka et al., 2022). Late-night digital messages, flirtatious pressure through social media, and emotional manipulation by coaches also fall within this scope.

Studies conducted in the Netherlands, Belgium, Germany, and Scandinavian countries have shown that 20% to 40% of elite female athletes have experienced sexual harassment at some point in their careers (Ohlert et al., 2020; Vertommen et al., 2017). However, such incidents are often underreported, with victims remaining silent due to fear of losing their sports careers.

These experiences not only constitute personal traumas but also reflect institutional neglect and broader gender inequality. Frequently taking place behind closed doors and remaining hidden, such incidents represent a form of invisible violence in sports. For example, in Canada's ice hockey leagues, it has been reported that coaches attempted to legitimize sexual demands from young female athletes as “rewards for success” (Mountjoy et al., 2020). Similarly, a 2020 scandal in the French athletics community revealed that several former national athletes had been systematically manipulated and abused over many years by the same coach. These cases starkly highlight the prevalence of institutional silence, a culture of impunity, and victim-blaming mentalities in sports (Kerr et al., 2020; Kavanagh et al., 2023). In this context, making sexual harassment against female athletes visible and creating safe spaces in sports is an urgent necessity.

In the context of the Turkish Republic of Northern Cyprus (TRNC), no comprehensive scientific study has yet explored female athletes‘ perceptions and experiences regarding sexual harassment. Therefore, this study aims to investigate female athletes' perceptions of sexual harassment, the extent to which they are exposed to it, and the need for safe environments in sports.

## Methods

This study was carried out using a descriptive research design, which is one of the commonly used research methods (Reviewer 3 and 4). This study focused on the perception dimension of the participants (Reviewer 4). The study population consisted of actively licensed female athletes over the age of 18 who participated in team sports. The sample was determined using purposive sampling. Of the 215 participants, 45.6% (*n* = 98) were football players, 23% (*n* = 51) basketball players, 17.2% (*n* = 37) volleyball players, and 13.5% (*n* = 29) handball players. Data were collected in person from female athletes by visiting sports clubs. Prior to administering the scales, informed consent forms were signed by the participants to indicate their voluntary participation. The study was approved by the Ethics Committee of the Graduate School of Social Sciences at Near East University with decision number 2024/1885 on November 18, 2024.

### Research question

Are there any differences in participants' reactions to sexual harassment, their attitudes toward sexual harassment, their perceptions of coaches' sexual harassment, and their gender role stereotypes according to their sociodemographic characteristics?

### Socio-demographic information form

In the study, a socio-demographic information form prepared by the researcher was used to collect personal data from the participants, such as age, type of sport, and the duration of participation in licensed sports.

### Coach behavior list

To measure the perception of sexual harassment, the study used the CBL developed by Auweele et al. (2008). The measurement tool was adapted into Turkish by Zengin (2012). As a result of the Turkish adaptation, it was determined that the instrument consisted of two subscales: sexual behaviors and non-instructional/potentially threatening behaviors. The Cronbach's alpha coefficient of the sexual behaviors subscale was 0.93, that of the non-instructional/potentially threatening behaviors subscale was 0.85, and the overall Cronbach's alpha for the scale was 0.89.

### Reactions to sexual harassment in sports

To assess the reactions of female athletes to sexual harassment in sports, the RSHS scale developed by Zengin (2012) was used. The instrument consists of 25 items and three subscales: emotional reactions, behavioral reactions, and passive reactions. The Cronbach's alpha coefficient for the overall scale was 0.79, while the coefficients were 0.84 for emotional reactions, 0.81 for behavioral reactions, and 0.60 for passive reactions.

### Ambivalent sexism inventory

Developed by Glick and Fiske (2018) to measure stereotypical attitudes toward gender roles, this instrument assesses hostile sexism and benevolent sexism. It was adapted into Turkish by Sakalli-Ugurlu (2002). The Turkish adaptation revealed that the instrument, consistent with the original, consists of 22 items and two subscales: hostile sexism and benevolent sexism. The Cronbach's alpha coefficient was 0.89 for the entire scale, 0.86 for the hostile sexism subscale, and 0.84 for the benevolent sexism subscale.

### Attitudes toward sexual harassment scale

To assess female athletes' attitudes toward sexual harassment, the study used the ATSHS developed by Sakalli-Uǧurlu (2010). The scale includes two subscales: attitudes that perceive sexual harassment as a consequence of provocative behaviors by women (ASHPBW) and attitudes that perceive sexual harassment as a trivial matter (ASHTM). The Cronbach's alpha coefficient for the overall scale was 0.83, while it was 0.90 for the ASHPBW subscale and 0.81 for the ASHTM subscale.

## Results

Upon examining Table 1, it is observed that there is no statistically significant difference in the total and subscale scores of the CBL among female athletes across different age groups (*p* > 0.05).

**Table 1:** Comparison of female athletes' coach/trainer behavior list scores by age group.

**Behavior list**	**Age group**	***n***	** $\bar{x}$ **	**s**	**Min**	**Max**	**F**	***p***
Sexual behaviors	18–21 years	91	9.92	1.27	9	13	2.887	0.058
	22–25 years	72	9.50	0.89	9	13		
	26–29 years	52	9.67	1.17	9	13		
Non-instructional/ potentially threatening behaviors	18–21 years	91	14.38	5.08	9	28	1.662	0.192
	22–25 years	72	13.36	5.42	9	28		
	26–29 years	52	12.90	4.30	9	28		
Coach behavior scale	18–21 years	91	24.31	5.65	18	41	2.107	0.124
	22–25 years	72	22.86	5.87	18	41		
	26–29 years	52	22.58	5.01	18	41		

According to Table 2, there are statistically significant differences in the total scores and the subscale scores (sexual behaviors and non-instructional/potentially threatening behaviors) of the CBL among female athletes based on their sport branches (*p* < 0.05). It was observed that handball players exhibited higher total and subscale scores in sexual behaviors and non-instructional/potentially threatening behaviors on the CBL compared to female athletes in football, basketball, and volleyball.

**Table 2:** Comparison of female athletes' coach behavior list scores by sport branch.

**Behavior list**	**Branch**	***n***	** $\bar{x}$ **	**s**	**Min**	**Max**	**F**	***p***	**Difference**
Sexual behaviors	Football	98	9.79	1.20	9	13	4.530	0.004^*^	1–4
	Basketball	51	9.80	1.34	9	13			2–4
	Volleyball	37	9.97	0.90	9	11			3–4
	Handball	29	9.03	0.19	9	10			
Non-instructional/potentially threatening behaviors	Football	98	14.96	5.07	9	28	6.666	0.000^*^	1–4
	Basketball	51	13.20	4.27	9	28			2–4
	Volleyball	37	13.49	5.72	9	26			3–4
	Handball	29	10.48	3.67	9	26			
Coach behavior scale	Football	98	24.74	5.63	18	41	7.174	0.000^*^	1–4
	Basketball	51	23.00	4.91	18	41			2–4
	Volleyball	37	23.46	6.33	18	37			3–4
	Handball	29	19.52	3.70	18	35			

^*^p < 0.05.

According to Table 3, there are statistically significant differences in the total and non-instructional/potentially threatening behavior subscale scores of the CBL among female athletes based on the duration of licensed sport participation (*p* < 0.05). Athletes who had been engaged in sports for 7 years or more had higher total and subscale scores in non-instructional/potentially threatening behavior on the CBL compared to those with 1–3 years and 4–6 years of experience.

**Table 3:** Comparison of female athletes' coach behavior list scores by duration of licensed sport participation.

**Behavior list**	**Duration**	***n***	** $\bar{x}$ **	**s**	**Min**	**Max**	**F**	***p***	**Difference**
Sexual behaviors	1–3 years	80	9.94	1.29	9	13	2.477	0.086	
	4–6 years	75	9.55	1.11	9	13			
	7 years and above	60	9.65	0.92	9	12			
Non-instructional/potentially threatening behaviors	1–3 years	80	15.14	5.10	9	28	8.368	0.000^*^	1–3
	4–6 years	75	13.69	5.64	9	28			2–3
	7 years and above	60	11.73	3.24	9	26			
Coach behavior scale	1–3 years	80	25.08	5.52	18	39	7.968	0.000^*^	1–3
	4–6 years	75	23.24	6.43	18	41			2–3
	7 years and above	60	21.38	3.66	18	38			

^*^p < 0.05.

There was no statistically significant difference in the sexual behaviors subscale scores of the CBL according to the duration of licensed sport participation (*p* > 0.05).

Upon examining Table 4, it is seen that there are no statistically significant differences in the scores obtained from the emotional, behavioral, and passive reaction subscales of the RSHS across age groups (*p* > 0.05).

**Table 4:** Comparison of female athletes' reactions to sexual harassment in sports scale scores by age group.

**Reactions**	**Age group**	***n***	** $\bar{x}$ **	**s**	**Min**	**Max**	**F**	***p***
RSHS-emotional reactions	18–21 years	91	28.33	13.19	10	50	0.879	0.417
	22–25 years	72	25.97	11.85	10	50		
	26–29 years	52	26.19	11.76	10	50		
RSHS-behavioral reactions	18–21 years	91	45.90	9.54	11	55	0.290	0.749
	22–25 years	72	45.56	9.87	23	55		
	26–29 years	52	46.85	8.89	23	55		
RSHS-passive reactions	18–21 years	91	7.80	4.44	4	20	0.629	0.534
	22–25 years	72	8.61	4.75	4	20		
	26–29 years	52	8.23	4.61	4	20		

According to Table 5, there is a statistically significant difference in the scores obtained from the emotional reactions subscale of the RSHS based on athletes' sports branches (*p* < 0.05). Female athletes in football and basketball received higher scores on the emotional reactions subscale of the RSHS compared to the athletes in the handball branch.

**Table 5:** Comparison of female athletes' reactions to sexual harassment in sports scale scores by sport branch.

**Reactions**	**Branch**	***n***	** $\bar{x}$ **	**s**	**Min**	**Max**	**F**	***p***	**Difference**
RSHS-emotional reactions	Football	98	28.59	12.64	10	50	2.968	0.033^*^	1–4
	Basketball	51	24.82	12.03	10	50			3–4
	Volleyball	37	29.51	13.44	10	46			
	Handball	29	22.41	9.19	10	46			
RSHS-behavioral reactions	Football	98	47.00	7.77	27	55	2.517	0.059	
	Basketball	51	43.86	10.91	11	55			
	Volleyball	37	48.19	8.65	23	55			
	Handball	29	43.69	11.98	23	55			
RSHS-passive reactions	Football	98	7.73	4.63	4	20	1.540	0.205	
	Basketball	51	9.25	4.75	4	20			
	Volleyball	37	7.57	4.57	4	18			
	Handball	29	8.55	3.96	4	16			

^*^p < 0.05.

There is no statistically significant difference in the scores on the behavioral and passive reactions subscales of the RSHS among female athletes in different branches (*p* > 0.05).

As shown in Table 6, there is a statistically significant difference in the emotional reactions subscale scores of the RSHS according to the athletes' duration of licensed sport participation (*p* < 0.05). Athletes with 7 or more years of licensed experience scored lower on the emotional reactions subscale of the RSHS than those with 1–3 years of experience.

**Table 6:** Comparison of female athletes' reactions to sexual harassment in sport scale scores by duration of licensed sport participation.

**Reactions**	**Duration**	***n***	** $\bar{x}$ **	**s**	**Min**	**Max**	**F**	***p***	**Difference**
RSHS-emotional reactions	1–3 years	80	30.05	13.23	10	50	4.402	0.013^*^	1–3
	4–6 years	75	26.16	11.78	10	46			
	7 years and above	60	24.07	11.30	10	50			
RSHS-behavioral reactions	1–3 years	80	46.65	8.34	23	55	0.583	0.559	
	4–6 years	75	46.20	10.21	11	55			
	7 years and above	60	44.93	9.97	23	55			
RSHS-passive reactions	1–3 years	80	7.53	4.52	4	20	2.338	0.099	
	4–6 years	75	9.07	5.24	4	20			
	7 years and above	60	7.93	3.55	4	16			

^*^p < 0.05.

There is no statistically significant difference between the scores obtained from the behavioral reactions and passive reactions subscales of the RSHS based on the duration of licensed sport participation of the female athletes in the study (*p* > 0.05).

Table 7 shows that there is no statistically significant difference in the hostile sexism subscale scores of the ASI based on age groups among female athletes (*p* > 0.05).

**Table 7:** Comparison of female athletes' scores on the ambivalent sexism inventory by age groups.

**ASI Inventory**	**Age group**	***n***	** $\bar{x}$ **	**s**	**Min**	**Max**	**F**	***p***	**Difference**
ASI-hostile sexism	18–21 years	91	30.26	10.82	10	50	2.308	0.102	
	22–25 years	72	26.94	9.63	10	50			
	26–29 years	52	29.17	8.26	10	49			
ASI-benevolent sexism	18–21 years	91	40.11	14.45	11	64	5.009	0.007^*^	1–2
	22–25 years	72	33.83	14.69	11	64			2–3
	26–29 years	52	40.44	12.07	15	59			

^*^p < 0.05.

However, a statistically significant difference was found in the benevolent sexism subscale scores by age group (*p* < 0.05). Athletes aged 22–25 scored higher than those aged 18–21 and 26–29.

Table 8 indicates that there is no statistically significant difference in hostile sexism subscale scores of the ASI among female athletes based on their sport branches (*p* > 0.05).

**Table 8:** Comparison of female athletes' scores on the ambivalent sexism inventory by type of sport.

**ASI Inventory**	**Branch**	***n***	**x**	**s**	**Min**	**Max**	**F**	***p***	**Difference**
ASI-hostile sexism	Football	98	30.11	8.68	10	50	1.505	0.214	
	Basketball	51	29.22	10.10	10	50			
	Volleyball	37	27.08	12.11	10	49			
	Handball	29	26.48	10.20	12	50			
ASI-benevolent sexism	Football	98	41.95	12.23	11	64	7.390	0.000^*^	1–4
	Basketball	51	37.27	15.97	13	64			2–4
	Volleyball	37	36.30	11.76	11	59			3–4
	Handball	29	28.76	15.98	13	64			

^*^p < 0.05.

However, a statistically significant difference was found in benevolent sexism subscale scores by sport branch (*p* < 0.05). Female handball players scored lower on the benevolent sexism subscale compared to those in football, basketball, and volleyball.

Table 9 shows that there is no statistically significant difference in hostile sexism subscale scores of the ASI based on duration of licensed sport participation among female athletes (*p* > 0.05).

**Table 9:** Comparison of female athletes' scores on the ambivalent sexism inventory by duration of licensed sport participation.

**ASI Inventory**	**Duration**	***n***	** $\bar{x}$ **	**s**	**Min**	**Max**	**F**	***p***	**Difference**
ASI-hostile sexism	1–3 years	80	30.08	10.46	10	50	1.124	0.327	
	4–6 years	75	28.68	9.71	10	50			
	7 years and above	60	27.57	9.38	12	49			
ASI-benevolent sexism	1–3 years	80	42.30	12.58	11	64	9.754	0.000^*^	1–3
	4–6 years	75	38.48	14.87	11	64			2–3
	7 years and above	60	31.98	13.65	13	59			

^*^p < 0.05.

However, a statistically significant difference was found in the benevolent sexism subscale scores (*p* < 0.05). Female athletes with 7 or more years of licensed experience had lower benevolent sexism scores compared to those with 1–3 and 4–6 years of experience.

According to Table 10, the comparison of female athletes' scores on the ATSHS by age groups reveals no statistically significant differences in either the “perceiving it as a consequence of women's provocative behavior” or “perceiving it as a trivial matter” subscales (*p* > 0.05).

**Table 10:** Comparison of female athletes' scores on the attitudes toward sexual harassment scale by age group.

**Attitudes**	**Age group**	***n***	** $\bar{x}$ **	**s**	**Min**	**Max**	**F**	***p***
ATSHS-perceiving it as a consequence of women's provocative behavior	18–21 years	91	18.89	6.01	10	32	0.849	0.429
	22–25 years	72	17.69	5.88	10	32		
	26–29 years	52	18.31	5.41	10	30		
ATSHS-perceiving it as a trivial matter	18–21 years	91	15.14	6.67	10	31	2.528	0.082
	22–25 years	72	15.31	6.23	10	31		
	26–29 years	52	13.08	4.05	10	26		

According to Table 11, there is a statistically significant difference in the scores female athletes obtained from the subscales of perceiving sexual harassment as a consequence of women's provocative behavior and perceiving it as a trivial matter based on their sports branch (*p* < 0.05). The scores of handball players on the subscale of perceiving it as a consequence of women's provocative behavior are lower than those of football and volleyball players. Meanwhile, the scores of football and volleyball players on the subscale of perceiving it as a trivial matter are lower than those of basketball and handball players.

**Table 11:** Comparison of female athletes' scores on the attitudes toward sexual harassment scale by sport branch.

**Attitudes**	**Branch**	***n***	** $\bar{x}$ **	**s**	**Min**	**Max**	**F**	***p***	**Difference**
ATSHS-perceiving it as a consequence of women's provocative behavior	Football	98	18.53	5.38	10	32	4.200	0.007^*^	1–4
	Basketball	51	17.47	6.05	10	32			3–4
	Volleyball	37	20.81	5.83	10	30			
	Handball	29	16.14	5.92	10	32			
ATSHS-perceiving it as a trivial matter	Football	98	13.24	4.84	10	31	7.145	0.000^*^	1–2
	Basketball	51	16.47	6.96	10	31			1–4
	Volleyball	37	13.57	4.13	10	26			2–3
	Handball	29	17.93	7.93	11	31			3–4

^*^p < 0.05.

When Table 12 is examined, it is seen that there is a statistically significant difference in the scores obtained from the subscales of perceiving sexual harassment as a consequence of women's provocative behavior and perceiving it as a trivial matter based on the duration of licensed sports participation (*p* < 0.05). Athletes with 1–3 years of licensed sports experience scored higher on the subscale of perceiving it as a consequence of women's provocative behavior compared to those with 4–6 years of experience. Those with 7 years or more of experience scored higher on the subscale of perceiving it as a trivial matter compared to those with 1–3 years and 4–6 years of experience.

**Table 12:** Comparison of female athletes' attitudes toward sexual harassment scale scores by duration of licensed sport participation.

**Attitudes**	**Duration**	***n***	** $\bar{x}$ **	**s**	**Min**	**Max**	**F**	***p***	**Difference**
ATSHS-perceiving it as a consequence of women's provocative behavior	1–3 years	80	19.71	5.58	10	32	4.697	0.010^*^	1–2
	4–6 years	75	16.89	5.74	10	29			
	7 years and above	60	18.35	5.89	10	32			
ATSHS-perceiving it as a trivial matter	1–3 years	80	13.36	4.76	10	31	7.129	0.001^*^	1–3
	4–6 years	75	14.24	5.69	10	31			2–3
	7 years and above	60	17.05	7.26	10	31			

^*^p < 0.05.

## Discussion

This study examined female athletes' perceptions of coach behavior, their reactions to sexual harassment in sports, their ambivalent sexist tendencies, and their attitudes toward sexual harassment from a multidimensional perspective. The primary aim of the study was to determine the relationships among these variables and to assess manifestations of gender-based inequality in sports.

The findings show that there is no statistically significant difference in the total and subscale scores of the Coach Behavior List according to the age groups of female athletes. This finding indicates that, regardless of age group, female athletes share similar perceptions regarding the overall Coach Behavior List and its subscales. In other words, coaches‘ attitudes and behaviors are evaluated similarly by athletes regardless of their age. Horn (2008) stated that athletes' perceptions of coach behavior are largely influenced by the coach's communication style and leadership behaviors, while demographic factors such as age may have limited effects on these perceptions.

According to the findings, there were significant differences in the total and subscale scores of the Coach Behavior List based on the athletes' sports branch. Handball players reported lower scores in the subscales of sexual behaviors and non-instructional threatening behaviors, indicating less exposure to such conduct. This implies that coach-athlete interactions may differ in terms of cultural, structural, and communication dynamics depending on the type of team sport (Lang and Hartill, 2014). In more popular and competitive environments such as football, basketball, and volleyball, authoritarian coaching styles and gender-based power imbalances are reported to be more prevalent (Johansson et al., 2022). In this context, the higher scores of female football players compared to other branches may indicate a greater risk of abuse in such environments.

The study also found a significant difference in the scores on the non-instructional/potentially threatening behavior subscale of the Coach Behavior List based on the duration of licensed sports participation. This result shows that female athletes perceive not only sexually explicit behaviors but also psychological pressure and emotional manipulation as threatening. This finding highlights the impact of power asymmetries and authority-based approaches in the coach-athlete relationship on female athletes (Johansson and Lundqvist, 2017; Stirling, 2009).

Another finding of the study is that there was no significant difference between the subscale scores of the Reactions to Sexual Harassment in Sports Scale based on the participants' age group, suggesting that emotional, behavioral, or passive reactions to sexual harassment do not vary by age. This finding indicates that age alone may not be sufficient to determine responses to such sensitive issues, and that other variables such as personal experience, level of social support, or education may play a more significant role. Consistent with this study, Fasting and Brackenridge (2009) also found that responses to sexual harassment in sports did not differ based on age. Furthermore, the study findings show that, by sports branch, football and basketball players exhibit stronger emotional responses to sexual harassment compared to handball players. This may be due to the nature of contact sports, where the body is constantly in focus, leading female athletes to feel objectified. Previous studies have indicated that branches where the risk of sexualization of the female body is higher may lead to more intense emotional reactions (Parent and Fortier, 2018; Daniels, 2009). On the other hand, in branches like football, where a male-dominated structure is more prominent, the suppression of emotions by female athletes is emphasized as a potential result of a culture of silence (Kavanagh et al., 2017; Stirling, 2009).

A negative correlation was found between the duration of licensed sports participation among female athletes and their emotional reactions. Female athletes who had been licensed for 1–3 years exhibited higher levels of emotional reaction compared to those with 7 or more years of experience. This may be interpreted as a result of a desensitization process that develops over time. In line with the findings of this study, other studies have also indicated that prolonged exposure may lead individuals to normalize such behaviors (Fasting and Sand, 2015; Hartill, 2005). Additionally, motivations such as the desire to preserve one's professional identity or the fear of being pushed out of the system may contribute to a decrease in reactivity (Stirling, 2009; Brackenridge, 2001).

In the Ambivalent Sexism Inventory, the fact that female athletes in the 22–25 age group scored higher on protective (benevolent) sexism compared to other age groups suggests that this age range may have more deeply internalized positive discrimination toward women. This supports the notion that attitudes related to gender roles vary across developmental stages (Sakalli-Ugurlu, 2002; Glick and Fiske, 2018). In other words, benevolent sexism can be internalized in different ways depending on age, and the relationship with gender roles is continuously reproduced within developmental and cultural contexts.

Another finding of the study revealed that female handball players scored lower on benevolent sexism than athletes in other branches. This suggests that gender norms may be less influential in certain sports disciplines. Similarly, LaVoi and Baeth (2017) noted that in sports such as handball, where collective dynamics prevail and gender distinctions are relatively less pronounced, the perception of sexism may be weaker.

The results also showed that as the duration of participation in sports increased, the level of benevolent sexism decreased. Female athletes who had been engaged in sports for seven years or more scored lower on benevolent sexism than those with 1–3 years and 4–6 years of experience. This suggests that prolonged engagement in sports may enhance one's ability to assess gender inequality more rationally. Long-term exposure to sports may strengthen female athletes' capacity to advocate for their autonomy (LaVoi and Baeth, 2017; Walker and Sartore-Baldwin, 2013).

The lack of significant differences across age groups in the subscales of the Attitudes Toward Sexual Harassment Scale indicates that attitudes such as perceiving sexual harassment as the result of provocative behavior by women or as a trivial matter do not vary with age. This finding suggests that such attitudes are shaped more by cultural norms and societal values than by age (Petersen and Hyde, 2010; Sakalli-Uǧurlu, 2010).

Another finding revealed a statistically significant difference in the subscales of the Attitudes Toward Sexual Harassment Scale related to seeing harassment as the result of women's provocative behavior and perceiving it as a trivial matter. Female handball players scored lower in the tendency to explain harassment through women's behavior, while football and volleyball players exhibited this tendency to a greater extent. On the other hand, football and volleyball players were less likely to perceive the matter as trivial, whereas basketball and handball players were more inclined to do so. This contradictory pattern suggests that the cultural structures of different sports branches shape female athletes' attitudes toward sexual harassment (Brackenridge and Fasting, 2005; Vertommen et al., 2016).

According to the findings, a significant difference was found between the duration of licensed sports participation among female athletes and their scores on the subscales of the Attitudes Toward Sexual Harassment Scale regarding the perception of harassment as a result of women's provocative behavior and perceiving it as a trivial matter. As the duration of sports participation increased, female athletes‘ attitudes toward sexual harassment showed significant variation. Specifically, participants with 1–3 years of experience were more likely to perceive sexual harassment as a result of women's provocative behavior, whereas those with 7 or more years of experience were more likely to perceive harassment as a trivial matter. This suggests that different mechanisms, such as internalization or desensitization, may play a role depending on the level of experience. Newcomers to sports may be more inclined to internalize gender norms or may not yet have developed a critical perspective on power relations in sports, leading them to adopt more victim-blaming attitudes (Jacobs et al., 2021). Conversely, in more experienced athletes, defensive mechanisms such as the normalization or tolerance of harassment may develop. This finding suggests that athletes' attitudes can evolve over time and that this evolution may occur not only through increased awareness but also through desensitization.

## Conclusion

This study explored the multifaceted interplay between female athletes' perceptions of coaching behaviors, their responses to sexual harassment in sport, ambivalent sexist tendencies, and attitudes toward harassment. The findings indicate that demographic factors such as age exert limited influence, whereas contextual and structural variables—such as sport branch and years of participation—are more decisive in shaping perceptions and reactions. Differences between branches highlight how cultural norms, communication styles, and gendered power dynamics embedded within specific sports environments can either amplify or reduce risks of harmful behaviors.

The results also suggest that length of exposure to organized sport contributes to a process of adaptation. Newer athletes tend to react more strongly to harassment, whereas those with longer experience appear to normalize or trivialize such behavior, possibly as a coping strategy to preserve their athletic careers. This underscores the need for systemic interventions that prevent desensitization and instead empower athletes to maintain critical awareness and self-advocacy.

Furthermore, the findings regarding ambivalent sexism demonstrate how benevolent attitudes toward women can persist in sport, albeit with variations across age groups and disciplines. While some athletes internalize gendered expectations, longer participation appears to foster greater recognition of inequality. However, the persistence of attitudes linking harassment to women's “provocative” behavior shows that cultural narratives remain influential and require targeted educational efforts.

Taken together, these results highlight the enduring influence of gendered power structures in sport and the importance of proactive safeguarding policies. Coaches, administrators, and sporting institutions must prioritize transparent communication, gender-sensitive education, and the establishment of protective reporting mechanisms. By fostering environments where female athletes feel supported rather than silenced, sports systems can move toward greater equity and safety.

## Limitations

The research was conducted with female athletes from specific team sports, which may limit the generalizability of the findings to individual disciplines or other cultural contexts.

The sample distribution across sports branches was unequal; for example, while 98 football players participated, only 29 handball players were included. This imbalance may have influenced the comparative findings and reduced the statistical power of branch-related analyses.

The reliance on self-reported data introduces the possibility of social desirability bias, as participants may have underreported sensitive experiences such as harassment or discriminatory behavior.

While the study highlighted the influence of sports branch and duration of participation, other potentially relevant factors such as socioeconomic background, education level, or access to institutional support were not examined. Addressing these limitations in future research would provide a more comprehensive understanding of the mechanisms underlying gender inequality and harassment in sport.

The findings should also be considered within the cultural perception of sports in Turkish society. In the local context, football is predominantly constructed as a male sport and holds strong symbolic value, which may reinforce gender hierarchies. By contrast, volleyball and basketball are perceived as more gender-neutral, attracting both male and female athletes under relatively equal conditions. Handball, however, is less visible and often considered a marginal sport, which may affect both participation rates and public attitudes. These cultural framings suggest that the differences observed in this study may not only stem from coach–athlete interactions, but also from the broader meanings assigned to each sport in our cultural setting.

## Data Availability

The original contributions presented in the study are included in the article/supplementary material, further inquiries can be directed to the corresponding author.
